# Pseudotumor cerebri and ciprofloxacin: a case report

**DOI:** 10.1186/1752-1947-5-104

**Published:** 2011-03-16

**Authors:** Rajeev R Fernando, Niraj N Mehta, Morgan G Fairweather

**Affiliations:** 1 Department of Internal Medicine, University of Texas Health Science Center, 6431 Fannin Street, Houston, Texas 77030, USA

## Abstract

**Introduction:**

We present a case of ciprofloxacin-associated pseudotumor cerebri in a 22-year-old African American woman. Withdrawal of ciprofloxacin in our patient resulted in complete resolution of ciprofloxacin-associated pseudotumor, as evidenced by a normal neuro-ophthalmic examination and a cerebrospinal fluid opening pressure of 140 mmH20.

**Case presentation:**

A 22-year-old African American woman presented with a headache of two weeks duration, visual blurring and horizontal diplopia after starting ciprofloxacin for pyelonephritis. An ophthalmic examination revealed that she had left eye esotropia, and a picture of the fundus demonstrated bilateral disc swelling without spontaneous venous pulsations. Magnetic resonance imaging of the brain and a magnetic resonance venogram were normal. A diagnostic lumbar puncture demonstrated an elevated opening pressure of 380mmH2O in a supine position. Laboratory examinations, including a cerebrospinal fluid exam, were unremarkable.

**Conclusion:**

ciprofloxacin-associated pseudotumor can cause chronic disabling headache and visual complications. Therapy is sub-optimal, often symptomatic, insufficient and complicated by side effects. When ciprofloxacin-associated pseudotumor presents in an atypical population, an inciting agent must be suspected because prompt withdrawal of the agent may lead to complete resolution of symptoms and prevent recurrence of similar episodes.

## Introduction

Pseudotumor cerebri (PTC) is a condition of increased intracranial pressure (ICP) without clinical, laboratory or radiological evidence of intracranial pathology. PTC is traditionally a disease of middle-aged, obese women and presents with symptoms of raised ICP, such as headache, pulsatile tinnitus, transitory visual obscurations and diplopia. PTC is a rare disease with increasing prevalence due to a global increase in the prevalence of obesity. The disease course is usually self-limiting, however some patients may experience chronic disabling headache and visual disturbances. Early intervention is necessary to prevent permanent visual defects from ischemic optic neuropathy. The pathophysiology of PTC is still not fully understood and so no real causal treatment exists. The treatment of PTC instead focuses on normalization of ICP, either medically or surgically. Both types of treatment are often symptomatic, insufficient and are complicated by side effects. Identifying a reversible cause may potentially reverse the disease and prevent future episodes.

## Case presentation

A 22-year-old African American woman presented with a two-week history of generalized, continuous headache, bilateral visual blurring and horizontal diplopia. With the persistence of the symptoms, as well as development of a left eye deviation, our patient was brought to our hospital. She was admitted two weeks prior to an outside hospital and empirically treated for pyelonephritis with 500 mg of ciprofloxacin twice daily for two weeks. Symptoms began two days after starting ciprofloxacin and worsened with continued treatment. She did not have a history of recent ear infection, treatment for acne, vitamin A supplementation, lead exposure, seizure disorder, oral contraceptives, steroid intake or withdrawal. There was no history of tick bites, target rash or joint pains. Her medical, surgical, travel and family history were all unremarkable.

**Figure 1:**
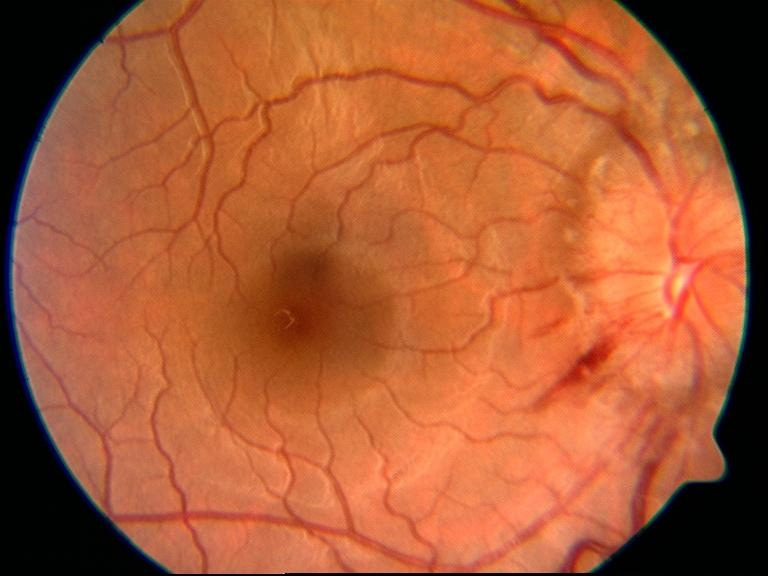
**Optic disc swelling after the initiation of ciprofloxacin**.

On examination, our patient's body mass index was 29.4. Her temperature was 37.2°C; heart rate 66/min, respiration rate 16/min, blood pressure 119/74 mmHg. On ophthalmic examination, her corrected visual acuity was 20/20 in her right eye and 20/40 in her left eye. She had diplopia on her left lateral gaze with left eye esotropia. The intra-ocular pressure, anterior segment and papillary response were normal. The fundus picture demonstrated bilateral disc swelling without spontaneous venous pulsations [Figure 1]. Both of her tympanic membranes were intact, and there was no evidence of bulging or discharge. She did not have neck rigidity and Kernig and Brudzinski signs were negative. Cranial nerves II to XII were intact. Manual muscle testing in all extremities was 5/5, and deep-tendon reflexes were all 2/4. Her cerebellar function was intact, as was gait, and Babinski reflexes were downward bilaterally. Sensation was intact to pain and soft touch. Findings of an examination of her lungs, heart, and abdomen were benign. Magnetic resonance imaging of her brain with and without contrast and a magnetic resonance venogram were normal. A diagnostic lumbar puncture performed in the recumbent position demonstrated an opening pressure of 380 mmH_2_O. Her cerebrospinal fluid (CSF) protein, glucose, cell count, veneral disease research laboratory test (VDRL), herpes simplex virus 1 and 2, *Toxoplasma*, cryptococcal antigen, Gram stain, acid fast bacilli stain, routine bacterial, viral, and fungal cultures, and cytology were all normal. CSF protein electrophoresis was normal without any oligoclonal bands. Laboratory data, including a complete blood count, chemistry panel (blood urea nitrogen of 16 mg/dL and creatinine of 0.8 mg/dL), liver function tests, thyroid function tests, sedimentation rate, anti-nuclear antibodies, VDRL, rheumatoid factor, and angiotensin-converting enzyme, were all normal. A urine beta human chorionic gonadotropin test was negative.

**Figure 2:**
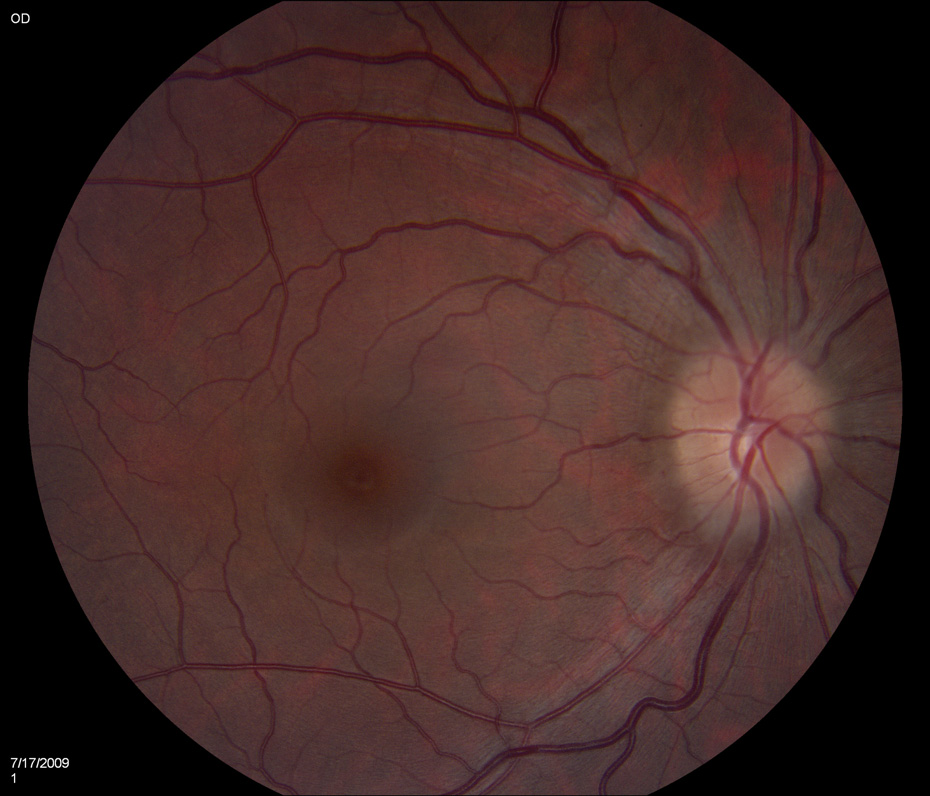
**Resolution of papilledema after the withdrawal of ciprofloxacin**.

A diagnosis of PTC was made. Ciprofloxacin was discontinued and serial lumbar punctures returned the opening pressure to 140 mmH_2_O. Our patient's symptoms improved after each lumbar puncture. The improvement of symptoms and subsequent reduction in CSF opening pressure was consistent with the elimination half-life of the drug. She was discharged home with close ophthalmology follow-up. Four weeks later, there was complete resolution of papilledema [Figure 2].

## Discussion

PTC has a rare, known association with members of the fluoroquinolone family of drugs and no reported association with pyelonephritis. It was first reported with nalidixic acid [1,2], the prototype drug of quinolone antibiotics. In 1966, a nine-year-old girl, hospitalized after an automobile injury resulting in multiple fractures, developed minimal papilledema while receiving nalidixic acid for a urinary tract infection; papilledema disappeared when the drug was discontinued. In 1967, the first reported case of nalidixic acid-associated pseudotumor cerebri was reported [2]. Symptoms recurred with rechallenge of nalidixic acid on two separate occasions and subsided with discontinuation [2,3]. Plasma nalidixic acid levels paralleled symptoms but were not elevated [2]. Since then ofloxacin [4], pefloxacin [5] and levofloxacin [6] have been associated with pseudotumor cerebri as well as one prior case of ciprofloxacin-induced disease [7]. Most cases have occurred in children and young adults. Disease has also been associated with non-fluoroquinilone antibiotics such as tetracycline [8] minocycline [9], and doxycycline [10]. Ciprofloxacin is a derivative of the drug nalidixic acid, with similar structural properties. The close temporal relationship between the use of ciprofloxacin and development of PTC, and the resolution of PTC after medication withdrawal, are only suggestive of a causal relationship. The exact pathogenesis of PTC from fluoroquinolones remains unclear.

## Conclusion

Persistent headache and visual symptoms after treatment with ciprofloxacin should prompt evaluation for pseudotumor cerebri. Increased awareness of this adverse effect is important, as ciprofloxacin is a widely used antibiotic for many infections. This case illustrates that despite the rarity of ciprofloxacin-induced PTC, patients and physicians should be aware of this possibility.

## Abbreviations

CSF: cerebrospinal fluid; ICP: intracranial pressure; PTC: pseudotumor cerebri; VDRL: veneral disease research laboratory.

## Consent

Written informed consent was obtained from the patient for publication of this case report and any accompanying images. A copy of the written consent is available for review by the Editor-in-Chief of this journal.

## Competing interests

The authors declare that they have no competing interests.

## Authors' contributions

MGF was involved in the management of the patient and initiated the preparation of the manuscript. RRF performed the literature search and was a contributor in writing the manuscript. NNM was also a contributor involved in editing the manuscript. All authors read and approved the final manuscript.
